# Research productivity, collaboration patterns, and socio-economic correlates of rheumatic fever and rheumatic heart disease research in Southeast Asia: a bibliometric analysis

**DOI:** 10.1186/s41043-026-01354-2

**Published:** 2026-06-10

**Authors:** Maria Llaine J. Callanta, Patrick R. Relacion

**Affiliations:** 1 Department of Biochemistry and Molecular Biology, College of Medicine, University of the Philippines - Manila, Pedro Gil St, Ermita Manila City, 1000 Philippines; 2 College of Public Health, University of the Philippines - Manila, Pedro Gil St, Ermita Manila City, 1000 Philippines; 3 College of Allied Health Professions, University of the East Ramon Magsaysay Medical Center, Aurora Blvd., Doña Imelda, Quezon City, 1113 Philippines

**Keywords:** Bibliometrics, Citation analysis, Rheumatic heart disease, Cardiology

## Abstract

**Background:**

Rheumatic fever and rheumatic heart disease (RF/RHD) remain important causes of preventable cardiovascular morbidity in low- and middle-income settings, including Southeast Asia. Although research output on these conditions has increased globally, the extent, thematic focus, and distribution of research productivity within Southeast Asia have not been comprehensively characterized. This study aimed to examine the publication trends, collaboration patterns, funding distribution, thematic focus, and selected socio-economic correlates of rheumatic fever and rheumatic heart disease research in Southeast Asia using bibliometric methods.

**Methods:**

Original articles and conference papers on RF/RHD in SEA from 1900 to 2024 were identified using the Scopus database. Bibliometric indicators and citation data were extracted and summarized descriptively. Country collaboration networks and author keyword co-occurrence maps were visualized using VOSviewer. Associations between selected country-level socio-economic indicators and bibliometric measures were explored using correlation analysis.

**Results:**

A total of 333 records on RF/RHD published between 1900 and 2024 from various SEA countries were obtained. Regional publication output remained relatively high in recent years, although annual counts fluctuated and the 2024 output was incomplete at the time of search. Thailand, Indonesia, Malaysia, Singapore, and the Philippines accounted for the largest share of regional output. However, despite this positive trend, SEA’s contribution to the global output for RF/RHD research is still below 2.5%. Notably, only one-fifth of the regional publications reported funding support, with marked disparities across countries. The retrieved literature was concentrated in themes related to diagnosis, surgery and complications, registries and epidemiology, anticoagulation, and valvular assessment. In contrast, topics such as social determinants of health, cost-effectiveness, implementation research, and patient-centered care appeared less represented. Country-level research productivity showed positive associations with selected socio-economic indicators and international collaboration, although these findings should be interpreted cautiously.

**Conclusion:**

Research on RF/RHD in Southeast Asia has grown, but publication output and funding remain unevenly distributed across countries. Continued efforts to strengthen international collaboration and research support are needed to amplify research productivity in low-resource Southeast Asian settings.

**Table:** 

**What is known in RF/RHD research?** Rheumatic fever and rheumatic heart disease remain important causes of preventable cardiovascular morbidity in Southeast Asia, particularly in low- and middle-income settings. Despite this continuing burden, research output from the region remains underrepresented in the global literature. Existing studies suggest that international collaboration may be associated with stronger research productivity and visibility, while much of the regional literature has focused on clinical and epidemiologic topics such as diagnosis, complications, surgery, and registries. **What new message this study addresses?** This study provides a bibliometric profile of rheumatic fever and rheumatic heart disease research in Southeast Asia across more than a century of publication. It quantifies regional publication output, collaboration patterns, funding representation, and thematic concentration, while identifying disparities across countries. The findings show that research productivity and indexed funding are unevenly distributed, with several Southeast Asian countries contributing little or no visible output in the retrieved literature. The study also highlights underrepresented research areas, particularly community-based prevention, implementation research, policy translation, social determinants of health, and patient-centered approaches. These findings provide a clearer regional evidence map that may help inform future research prioritization and capacity-building efforts.	

## Introduction

Acute rheumatic fever (ARF) is an autoimmune disease affecting various organs following uncontrolled Group A Streptococcal (GAS) infection. In the heart, autoimmune antibodies against cardiac myocytes are formed as a result of molecular mimicry to certain GAS proteins leading to increased cardiac tissue inflammation and damage[1]. Repeated and uncontrolled ARF may lead to the development of rheumatic heart disease (RHD), the most significant cardiovascular complication causing mortality to young adults [2]. In fact, among the ARF patients, 35·9% progresses to RHD within one year [3] and within the first decade, majority (61%) may develop to RHD [4]. The global prevalence and incidence of RHD in children have increased by 40·88% and 41·89%, respectively from 1990 to 2019 [5]. RHD is also a leading cause of heart failure among children and young adults worldwide, with estimated 306,000 deaths each year [6]. Although global mortality from RHD is projected to decline by 2030, RHD-associated heart failure is expected to increase [7]. In Asia, approximately 22·24 million were reported to have RHD, with 249,830 deaths in 2019. While many Asian countries showed a declining mortality and a decreasing age-standardized years of life lost between 1990 and 2019, Southeast Asia (SEA) demonstrated an increasing trend in both age-standardized incidence rate and age-standardized prevalence rate [8]. In the Philippines, for example, the annual average percentage change for age-standardized incidence rate and age-standardized prevalence rate was 2.0% and 1.5%, respectively, with a recorded RHD-associated death rate of 1.09 per 100,000 population. Nevertheless, available local data remain limited [9]. The burden of RHD in SEA continues to be concentrated in low-income settings and in marginalized or economically deprived communities within middle- and high-income countries.

Clinical manifestation of RHD involves progressive cardiac valve dysfunction exacerbated by thromboembolism, arrhythmia, and cardiac failure. Current strategies to reduce GAS-related post-infectious sequelae include anti-inflammatory treatment during acute rheumatic fever and long-term secondary prophylaxis to prevent recurrence and delay disease progression [3]. However, the delivery and long-term implementation of these strategies remain challenging in many resource-limited settings because of barriers related to access, adherence, cost, and continuity of care [2]. These persistent clinical and health-system challenges underscore the continued need for a strong and regionally relevant evidence base for RF/RHD prevention, diagnosis, management, and control.

Over the last two decades, interest in RF/RHD research has increased, partly because of wider use of echocardiography for the detection of previously unrecognized disease and growing recognition of the global burden of RHD [10, 11]. However, the research landscape in SEA has not been comprehensively characterized despite the region’s continuing disease burden and heterogeneity in health systems, resources, and research capacity. A clearer understanding of publication trends, major research themes, collaboration patterns, funding representation, and country-level disparities is needed to identify gaps and guide future research prioritization in the region. Bibliometric analysis is a quantitative approach used to examine publication output, citation patterns, collaboration networks, and thematic trends within a defined body of literature. Hence, this study aimed to characterize the research productivity, collaboration patterns, thematic focus, funding distribution, and selected socio-economic correlates of rheumatic fever and rheumatic heart disease research in Southeast Asia through bibliometric analysis.

## Methods

### Data source and search strategy

A bibliometric database search was conducted using the Scopus database. The search query used was: TITLE-ABS-KEY (“rheumatic feve*” OR “rheumatic heart diseas*” OR “Bouilla* diseas*”). Records were limited to English-language publications and to the following subject areas: Medicine, Biochemistry, Genetics and Molecular Biology, Nursing, Pharmacology, Toxicology and Pharmaceutics, Immunology and Microbiology, Multidisciplinary, Health Professions, and Dentistry. All searches were performed on August 5, 2024.

**Fig. 1:**
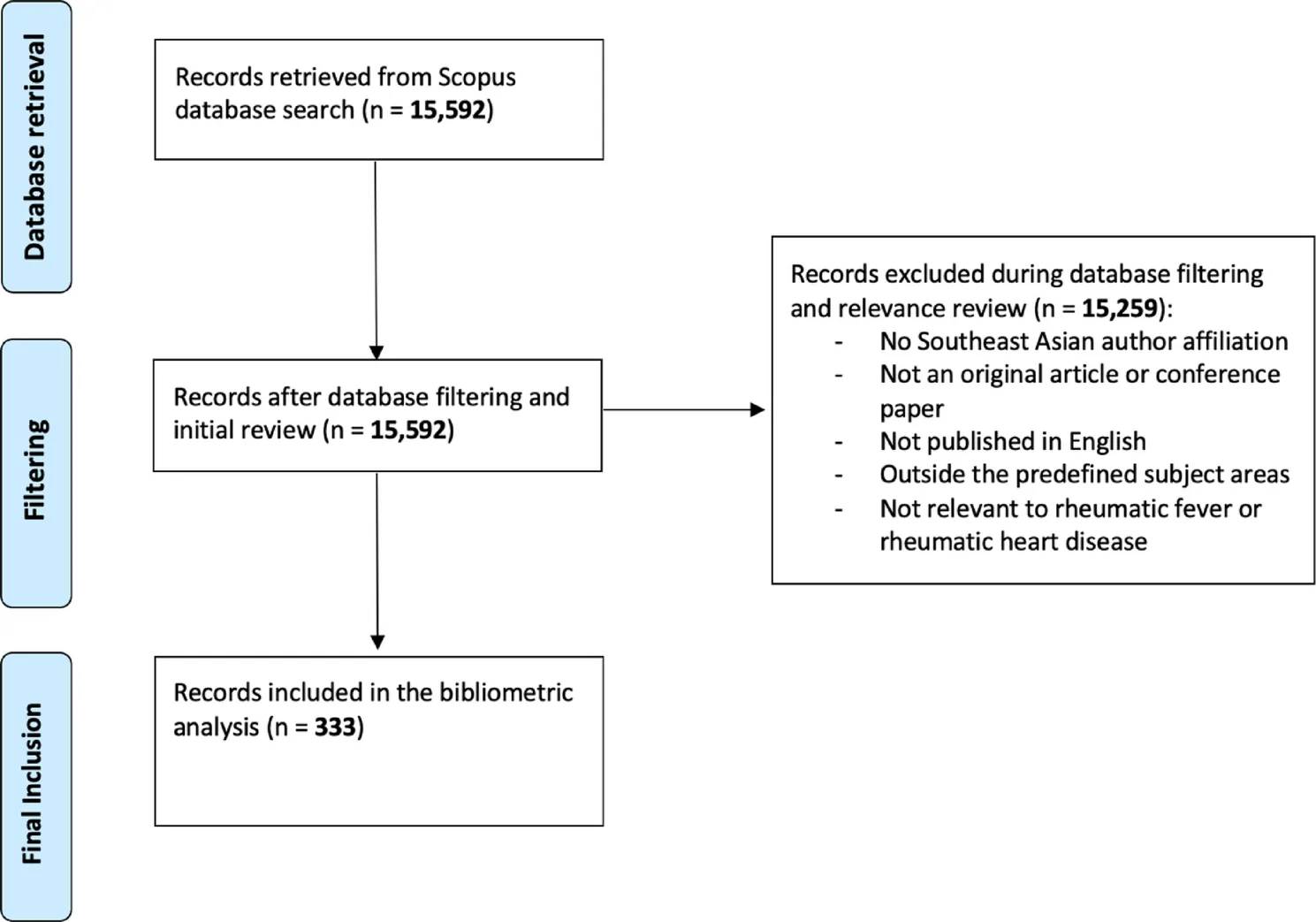
Flow diagram of record retrieval, filtering, and inclusion for the bibliometric analysis of rheumatic fever and rheumatic heart disease research in Southeast Asia

For the purposes of this study, Southeast Asia attribution was based on author affiliation. Publications were included if at least one author was affiliated with a Southeast Asian country. Because a single publication may include authors from more than one Southeast Asian country, country-level publication counts were treated as non-mutually exclusive using affiliation-based full counting. Therefore, the sum of country-specific records exceeds the number of unique publications. Retrieved records were also reviewed for topical relevance, and false-positive records unrelated to rheumatic fever or rheumatic heart disease were excluded. The summary of the search is presented in Fig. 1.

### Data collection

Original articles and conference papers were retrieved from the Scopus database. For each record, the following bibliometric variables were extracted: authors, year of publication, title, journal, institution, country affiliation, author keywords, citation count, indexed funding information, and subject area. These variables were used to describe publication trends, country-level research productivity, collaboration patterns, funding representation, and thematic areas of RF/RHD research in Southeast Asia.

### Statistical analysis

Statistical analyses were performed using GraphPad Prism version 8 (GraphPad Software, San Diego, CA, USA). Associations between country-level characteristics and bibliometric indices were explored using Spearman’s rank-order correlation. Gross domestic product and gross domestic product per capita were obtained from the International Monetary Fund [12]. Research and development expenditure as a percentage of gross domestic product, physician-to-population ratio, and researcher-to-population ratio were obtained from the World Bank [13]. A two-sided P-value of less than 0.05 was considered statistically significant. Given the small number of country-level observations, these correlation analyses were treated as exploratory and interpreted cautiously.

Visualization of country collaboration networks and author keyword co-occurrence patterns was performed using VOSviewer version 1.6.16 (Leiden University, Leiden, the Netherlands) [14]. Network visualizations were interpreted descriptively to illustrate broad patterns of collaboration and thematic clustering within the retrieved literature. For the correlation analysis, international collaboration was operationalized as the number of country-level co-authorship links between a Southeast Asian country and countries outside Southeast Asia, as derived from the VOSviewer country co-authorship network. Meanwhile, local collaboration was operationalized as the number of country-level co-authorship links between a Southeast Asian country and other Southeast Asian countries. These indicators were used descriptively as proxies of collaboration visibility rather than as direct measures of collaboration quality or intensity.

## Results

### Annual number of Rheumatic fever and rheumatic heart publications

**Fig. 2:**
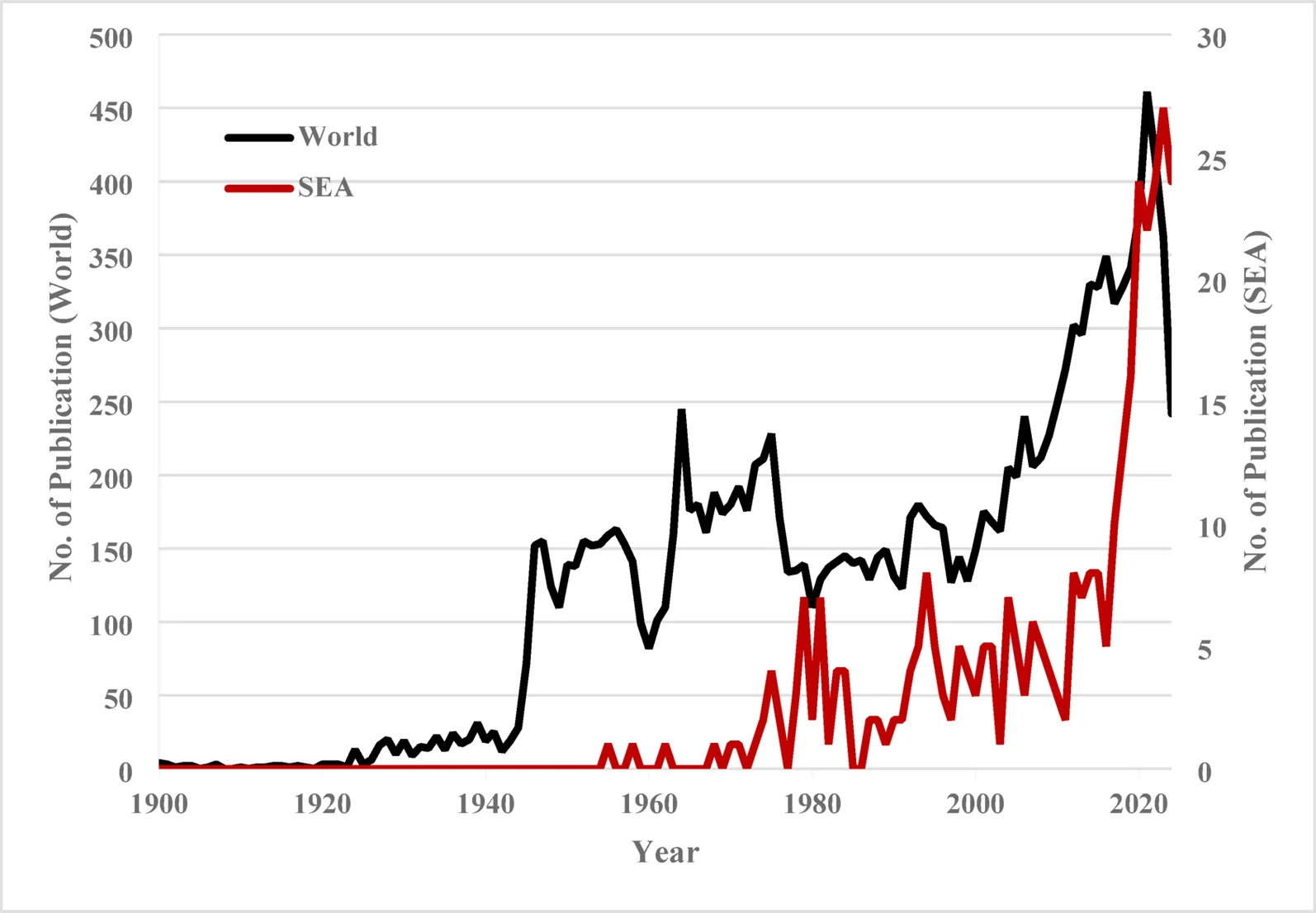
Annual number of publications on rheumatic fever and rheumatic heart disease worldwide and in Southeast Asia from 1900 to 2024

A total of 333 articles and conference papers on RF/RHD from Southeast Asia published between 1900 and 2024 were identified (Fig. 2). Publication activity from the region remained minimal during the early decades of the study period, with visible contributions emerging from Thailand, Malaysia, Singapore, the Philippines, and Indonesia beginning in the mid-twentieth century. Research output from Southeast Asia increased more noticeably from the 2000 s onward, with more than half of the retrieved records published within the last decade. Despite this upward trend, Southeast Asia accounted for only 2.1% of the total global literature retrieved on RF/RHD during the study period.

### Rheumatic fever and rheumatic heart disease publications by country

**Fig. 3:**
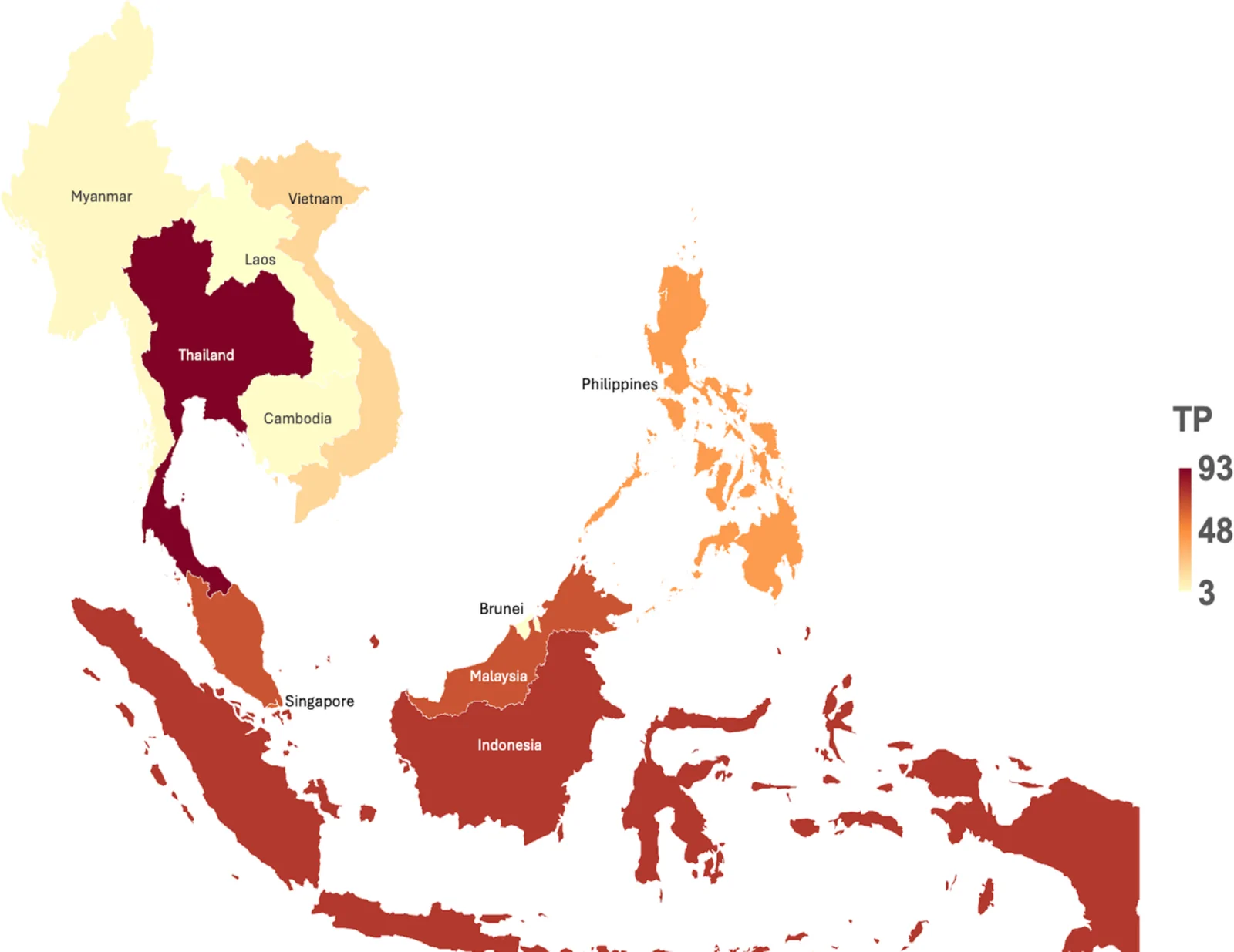
Country-level publication output on rheumatic fever and rheumatic heart disease research in Southeast Asia

Country-level productivity and citation metrics for RF/RHD research in Southeast Asia are summarized in Table 1. Using affiliation-based full counting, Thailand contributed the largest number of publications (*n* = 93, 24.41%), followed by Indonesia (*n* = 75, 19.69%), Malaysia (*n* = 66, 17.32%), Singapore (*n* = 60, 15.75%), and the Philippines (*n* = 42, 11.02%) (Fig. 3). In contrast, Cambodia, Brunei Darussalam, and Laos had the lowest publication counts, together accounting for a small proportion of the regional output.

In terms of total citations, Malaysia recorded the highest citation count (*n* = 12,513), followed by Singapore (*n* = 12,322) and the Philippines (*n* = 12,104). Thailand had the highest H-index (18), followed by Malaysia (16) and Singapore (14). Citation per publication values were notably high in several countries with relatively small publication counts, particularly Viet Nam and Myanmar. These values should be interpreted cautiously because country-level citation metrics were generated using affiliation-based full counting, such that highly cited multinational publications may contribute to the citation totals of more than one country.

**Table 1:** Citation analysis of rheumatic fever and rheumatic heart disease research published from Southeast Asian countries

Country	Total publications (TP)	Total citations (TC)	Citation per paper (CPP)	H-index
Thailand	93	1180	12·69	18
Indonesia	75	12,026	160·35	10
Malaysia	66	12,513	189·59	16
Singapore	60	12,322	205·37	14
Philippines	42	12,104	288·19	10
Viet Nam	19	11,486	604·53	9
Timor-Leste	10	104	10·40	6
Myanmar	6	2108	351·33	4
Cambodia	4	639	159·75	2
Laos	3	46	15·33	2
Brunei Darussalam	3	15	5·00	1

Country-level publication and citation metrics were generated using affiliation-based full counting. Therefore, a single publication with multiple Southeast Asian country affiliations could contribute to more than one country total. Citation per publication values in countries with small publication counts should be interpreted cautiously

**Fig. 4:**
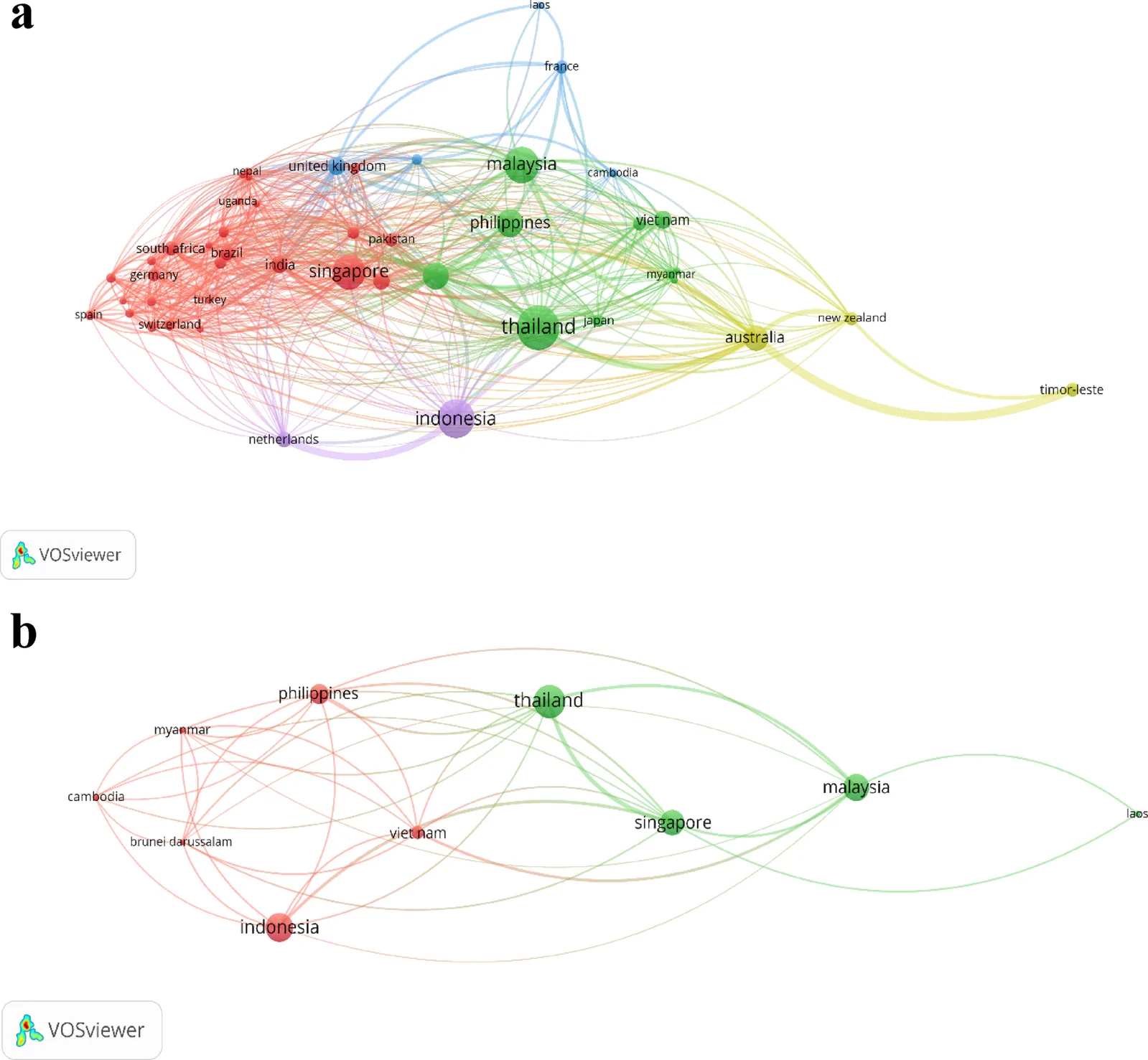
Network visualization of country collaboration in rheumatic fever and rheumatic heart disease research at the global level **(a)** and within Southeast Asia **(b)**. Node size is proportional to the number of links of each country in the collaboration network, while line thickness reflects the relative strength of the connection between countries

Network visualization showed that rheumatic fever and rheumatic heart disease research in Southeast Asia involved both intra-regional and international collaboration (Fig. 4). At the global level, several Southeast Asian countries were connected to collaborators outside the region, indicating that the regional literature has been shaped partly through cross-country partnerships. Thailand appeared as a central node in the global collaboration network and had the highest number of international linkages. Within Southeast Asia, collaboration links were also observed among multiple member countries, although the pattern was uneven across the region. Overall, countries with higher publication output, particularly Thailand, Malaysia, Singapore, and Indonesia, also occupied more prominent positions in the collaboration networks.

Indexed funding information by country is summarized in Table 2. Indonesia recorded the highest absolute number of funded publications (*n* = 17), representing 22.7% of its total RF/RHD output. Singapore followed with 16 funded publications (26.7%), while Thailand, despite having the highest total publication count, had 15 funded publications (16.1%). Timor-Leste had the highest proportion of funded publications, with 4 of 10 records (40.0%) indicating indexed funding support. In contrast, Myanmar, Laos, and Brunei Darussalam had no funded publications recorded in the retrieved dataset. Overall, only a minority of the regional literature reported indexed funding support, and notable disparities were observed across countries.

**Table 2:** Indexed funding representation in rheumatic fever and rheumatic heart disease publications from Southeast Asian countries

Country	Total number of funded studies	Total publications (TP)	Percentage (%)
Indonesia	17	75	22.7
Singapore	16	60	26.7
Thailand	15	93	16.1
Malaysia	13	66	19.7
Philippines	8	42	19.0
Timor-Leste	4	10	40.0
Viet Nam	1	19	5.3
Cambodia	1	4	25.0
Myanmar	0	6	0.0
Laos	0	3	0.0
Brunei Darussalam	0	3	0.0

### Rheumatic fever and rheumatic heart diasease publications by the institution

The leading institutions contributing to RF/RHD research in Southeast Asia were primarily based in Thailand, Indonesia, and Singapore. Mahidol University ranked first in publication output with 33 records, followed by Universitas Indonesia with 30 records and the National University of Singapore with 26 records. In terms of citation impact, the National University of Singapore had the highest citation count (*n* = 3,909) and the highest H-index (9) among the top contributing institutions.

### Rheumatic fever and rheumatic heart disease publications by journal

More than half of the ten most productive journals in RF/RHD research from Southeast Asia were based within the region. Among these, two journals were based in Singapore, while one each was based in Thailand, Malaysia, the Philippines, and Indonesia. The Journal of the Medical Association of Thailand published the largest number of Southeast Asia-affiliated RF/RHD records (*n* = 23), whereas Southeast Asia-affiliated RF/RHD records published in The Lancet accumulated the highest citation count (*n* = 11,934).

### Rheumatic fever and rheumatic heart disease research trends in SEA

**Fig. 5:**
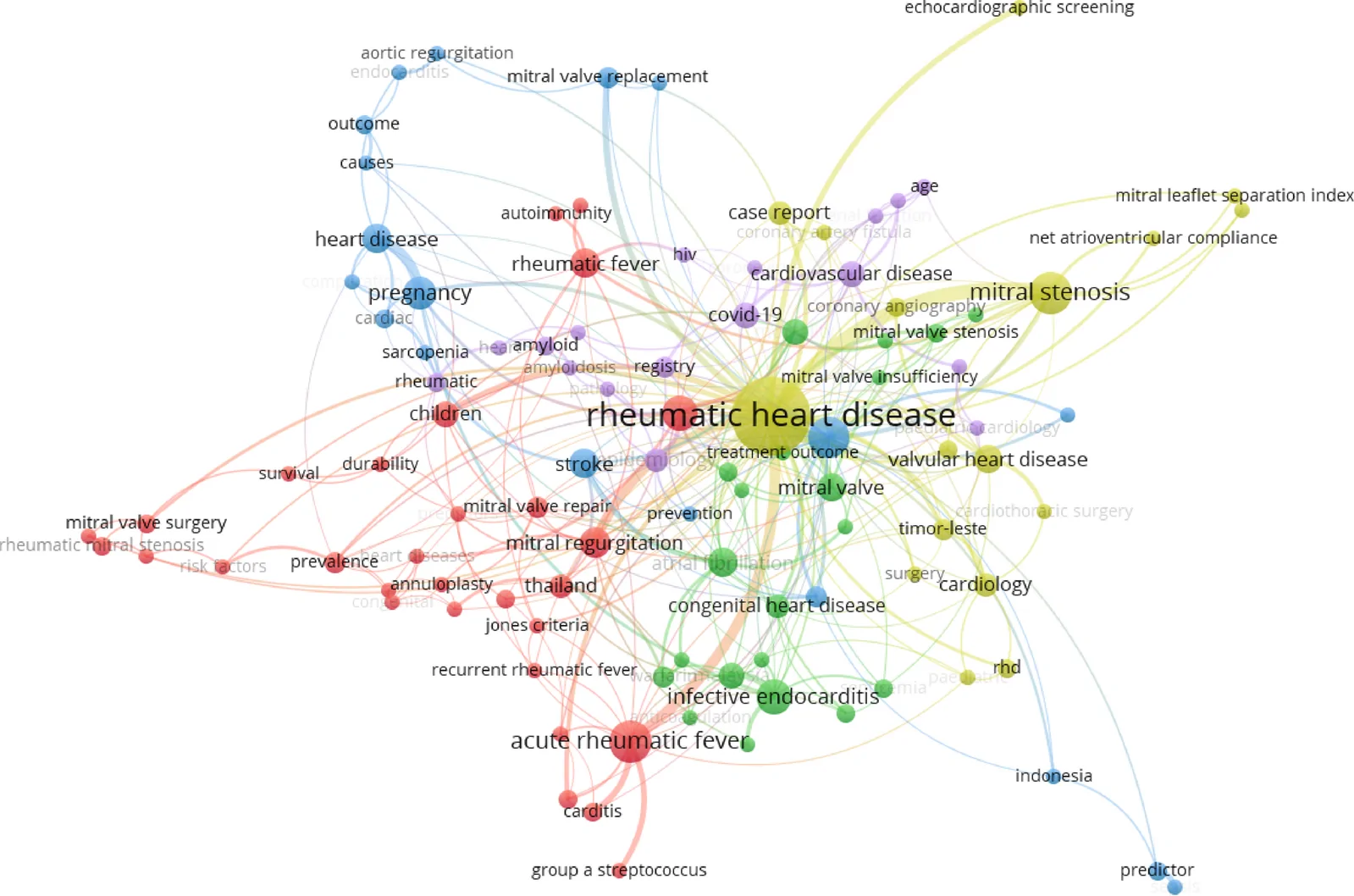
Network visualization of author keyword co-occurrence in rheumatic fever and rheumatic heart disease research in Southeast Asia from 1900 to 2024

Author keyword co-occurrence mapping was used to examine the thematic structure of RF/RHD research in Southeast Asia (Fig. 5). The network visualization showed five major keyword clusters, reflecting the main areas of emphasis in the retrieved literature. One cluster was centered on registry-related and epidemiologic terms. Another cluster was composed of keywords linked to acute rheumatic fever, diagnosis, Group A Streptococcus, Jones criteria, recurrent rheumatic fever, and risk-related terms. A third cluster was dominated by clinical management and complication-related keywords, including anticoagulation, infective endocarditis, mitral valve insufficiency, atrial fibrillation, and other treatment outcome terms. A fourth cluster included comorbidity- and outcome-related terms such as stroke, aortic regurgitation, pregnancy, heart disease, and sarcopenia. The fifth cluster was characterized by diagnostic and valvular assessment terms, including echocardiographic screening, coronary angiography, mitral leaflet separation index, net atrioventricular compliance, and mitral stenosis.

Overall, the keyword network suggests that the regional literature has largely focused on diagnosis, clinical complications, valvular pathology, treatment-related issues, and epidemiologic or registry-oriented work. In contrast, health systems research, implementation research, social determinants of health, and patient-centered care appeared less visible within the keyword landscape.

### Country-level socioeconomic correlates of rheumatic fever and rheumatic heart disease research productivity and impact

The correlations between selected country-level socio-economic indicators and bibliometric measures of RF/RHD research in Southeast Asia are shown in Table 3. Gross domestic product was positively correlated with total publications, total citations, and H-index. Research and development expenditure was also positively correlated with total publications and H-index. In addition, the number of researchers in research and development showed a positive correlation with H-index. International collaboration demonstrated positive correlations with all three bibliometric indicators, whereas intra-regional collaboration was positively correlated only with total citations.

Gross domestic product per capita and physician density were not significantly correlated with the bibliometric indicators examined. Given the small number of countries included and the ecological nature of the analysis, these findings should be interpreted cautiously and should not be regarded as evidence of causation.

**Table 3:** Correlations between selected country-level socio-economic indicators and bibliometric measures of rheumatic fever and rheumatic heart disease research in Southeast Asia

Indicators	Bibliometric indicators	*r*	*P* value
**Gross domestic product** **(in US dollars**,** billions)**	Total Publications	0·8428	**0·1900**
	Total Citations	0·6909	**0·0226**
	H-index	0·7671	**0·0079**
**Gross domestic product per capita** **(in US dollars)**	Total Publications	0·4328	0·1843
	Total Citations	0·3636	0·2731
	H-index	0·4429	0·1731
**Research and development expenditure (% of gross domestic product)**	Total Publications	0·6973	**0·0296**
	Total Citations	0·5976	0·0732
	H-index	0·7914	**0·0087**
**Researchers in research and development** **(per million people)**	Total Publications	0·6079	0·0679
	Total Citations	0·4303	0·2182
	H-index	0.6464	**0·0484**
**Physicians (per 1000 people)**	Total Publications	0·3777	0·25
	Total Citations	0·3955	0·2286
	H-index	0·5024	0·1173
**International collaboration**	Total Publications	0·8453	**0·0018**
	Total Citations	0·7051	**0·0185**
	H-index	0·8218	**0·0029**
**Local collaboration**	Total Publications	0·5118	0·1122
	Total Citations	0·6528	**0·0306**
	H-index	0·5236	0·1037

Bold *P* values denote statistical significance at *p* < 0.05

## Discussion

This bibliometric analysis characterized the research productivity, collaboration patterns, thematic focus, funding representation, and selected socio-economic correlates of RF/RHD research in Southeast Asia. Although records were retrieved as early as 1900, publication output from the region remained limited for much of the twentieth century, with more visible growth emerging only in recent decades [15]. This pattern should be interpreted with awareness that Scopus coverage of earlier literature was historically less complete, although retrospective expansion of indexed records has improved the usability of long-term publication trend analyses [16]. The present findings nevertheless show that the regional literature remained sparse for many decades before increasing more noticeably from the 2000s onward, with more than half of the retrieved records published within the last decade. This upward trajectory broadly mirrors the growing global visibility of RF/RHD as persistent but preventable causes of cardiovascular morbidity and mortality, particularly in low- and middle-income settings. The increase in publication activity in recent years may also have occurred alongside renewed international advocacy and prevention efforts, including the 2016 World Heart Federation roadmap, which helped strengthen global attention toward disease control and research prioritization [17].

Despite this growth, Southeast Asia still accounted for only a small share of the global literature retrieved on RF/RHD. Health research is resource-intensive and often requires sustained funding, trained personnel, infrastructure, and longitudinal follow-up. Without adequate national funding allocation, medical research may be delayed or constrained. In comparison to the top-ranking countries in RF/RHD research outputs such as the United States of America, the United Kingdom and China which allocate 3·46%, 2·91% and 2·43% of their GDP to research, respectively, most Southeast Asian countries allocate less than 1% of gross domestic product to research and development except Singapore, which allocates approximately 2.1% of gross domestic product to research and development. In the present study, gross domestic product and research and development expenditure were positively correlated with selected bibliometric indicators, although these associations should not be interpreted causally. These findings are nevertheless consistent with previous studies suggesting that stronger research investment environments are often associated with greater scientific productivity, implementation capacity, and innovation. [18]. Strengthening national and institutional investment in research and development may improve scientific productivity and support innovation [19, 20].

The regional output was led by Thailand, Indonesia, Malaysia, Singapore, and the Philippines, while Malaysia and Singapore showed the highest country-level citation counts and Thailand had the highest H-index. The collaboration networks further showed that higher-output countries generally occupied more central positions both within Southeast Asia and in the broader international network. This pattern is consistent with previous literature highlighting the role of collaborative work in strengthening expertise, technology sharing, and access to research resources [21]. At the same time, the present findings do not establish that collaboration itself causes higher productivity, since publication output, citation visibility, institutional capacity, and international partnerships are likely interrelated. National and institutional policies that incentivize publication may also contribute to these observed patterns. In Indonesia, in the hope to bolster the nation’s scientific culture, the government offered reward incentives to eligible professors who publish a specified number of manuscripts in locally-accredited or international journals within the last three years subject to re-evaluation every three years [22]. Similar publication incentive policies have been observed in other nations within the region [23, 24]. In other countries such as Viet Nam, publication of two (2) papers in international academic journals has been mandated as a graduation requirement for doctoral candidates [25].

The analysis of indexed funding information also showed marked disparities across countries. Indonesia, Singapore, and Thailand had the largest absolute numbers of funded publications, while Timor-Leste showed the highest proportion of funded records relative to its total output. These findings suggest uneven visibility of research support across the region. However, funding information in bibliometric databases should be interpreted cautiously because indexed funding fields may not fully capture all funding sources, institutional support, or collaborative resource-sharing arrangements. Thus, countries with low recorded funding may not necessarily have had no support, but rather limited indexed acknowledgement in the retrieved records. Aside from this, the absence or scarcity of funded studies in these nations may reflect limited national prioritization, weak institutional capacity, or constrained access to regional and international funding mechanisms. Addressing these disparities is critical to strengthening regional research equity. The availability of RF/RHD-specific grants can play a transformative role, not only in enhancing scientific output but also in empowering early-career researchers, enabling international research collaborations, and integrating evidence into national disease control policies.

Most highly productive journals in the retrieved dataset were regional or domestic journals, indicating that locally based publication venues continue to play an important role in disseminating RF/RHD research from Southeast Asia. Publishing in regional or domestic journals may offer practical advantages, including greater contextual relevance, shorter editorial timelines, and improved accessibility for local readers. Another factor to consider in publishing articles to international journals is English proficiency. Countries from English speaking nations have an increased chance of acceptance to Scopus-indexed papers compared with non-native English speakers [26]. This becomes a challenge for countries such as Thailand and Vietnam, where international publication has recently been included as a requirement for processing scientific grants [24, 27].

One major structural barrier that such funding could help mitigate is the prohibitive cost of article processing charges (APCs), especially in high-impact open-access journals [28]. In many Southeast Asian countries, institutional and government grants do not explicitly allocate funds for APCs, thereby limiting researchers’ ability to publish in venues that ensure global visibility and citation potential [29]. Consequently, high-quality research may remain unpublished or confined to local journals, diminishing its impact on regional and international health agendas. Without support mechanisms to cover APCs, even well-executed studies may remain inaccessible to the global scientific community. This underlines the need for regional and global research funders to implement APC waivers, develop publishing grants, and establish partnerships with journals to support equitable access to publication.

The author keyword network suggests that the regional literature has mainly concentrated on diagnosis, valvular pathology, clinical complications, treatment-related care, and registry or epidemiologic topics. This pattern is understandable given the persistent burden of advanced rheumatic heart disease, the importance of echocardiographic detection, and the ongoing need to manage valvular dysfunction, infective endocarditis, anticoagulation, and surgical outcomes. In other words, much of the visible literature reflects the downstream clinical consequences of disease that has already progressed. This emphasis is clinically important, but it also highlights a relative underrepresentation of other domains that are critical for long-term disease control. In particular, primary prevention, community-based intervention studies, implementation science, health systems research, social determinants of health, and patient-centered care appeared less visible in the keyword landscape. These areas are especially relevant in Southeast Asia, where disease burden is concentrated in underserved populations and where effective control depends not only on diagnosis and treatment, but also on continuity of prophylaxis, early detection, service integration, and access to care. Interventions should not be treated as one-size-fits-all because countries differ in resources, health-system capacity, and access to preventive and therapeutic services. Given that most cases occur in populations from socioeconomically disadvantaged settings, accessibility and availability of therapeutic options are necessary to lessen the overall burden of this condition. As reflected on the focus of most studies, there is a heightened push for wider coverage of benzathine penicillin treatment to patients at risk of RHD and its complications. An example is the RF/RHD outpatient package of the Philippine Department of Health, which is intended to provide free benzathine penicillin prophylaxis to diagnosed patients. On the other hand, in some low-income countries in Africa, school-based clinics were established to have an early detection and treatment system for GAS pharyngitis among school-aged children [30]. Integration of community-based health promotion programs against RF/RHD and establishment of partnership with the local health agencies or departments to generate surveillance registry and improved access to RF/RHD care have also been reported as effective strategies for improving disease management [31, 32]. Moreover, these programs and public health strategies can be applied to low-income countries in SEA to overcome the challenges in resources and budget allocations.

Findings of this study support the current trends in RF/RHD research in SEA and several gaps were identified. Future studies should place greater emphasis on primary prevention, community-based interventions, adherence to long-term secondary prophylaxis, health systems integration, implementation research, health economics, and policy translation. These areas are particularly important for countries with limited research output, where weak surveillance systems, fragmented care pathways, and constrained research infrastructure may limit the generation of locally actionable evidence. Strengthening these domains would help move the regional research agenda beyond description of disease burden toward prevention, service delivery, and sustainable control [33, 34].

This study has several limitations. First, the analysis included only Scopus-indexed records and may therefore have missed relevant publications indexed exclusively in other databases or local repositories. Second, country attribution was based on author affiliation using full counting, such that a single multinational publication could contribute to more than one country total; this may inflate country-level publication and citation metrics, particularly citation per publication values in countries with small denominators. Third, the correlation analyses were ecological, involved a small number of country-level observations, and were susceptible to outlier influence; accordingly, they should not be interpreted as causal. Finally, indexed funding information may not fully reflect all forms of research support.

## Conclusion

This bibliometrics analysis shows that RF/RHD research in Southeast Asia has increased over time, particularly in the last decade, but the region remains underrepresented in the global literature. Research output, citation visibility, and indexed funding were concentrated in a small number of countries, while several low-resource settings contributed little visible evidence in the retrieved database. The observed associations between research productivity, socioeconomic indicators, and collaboration should be interpreted as exploratory country-level patterns rather than causal relationships. The regional literature was concentrated mainly on diagnosis, clinical complications, valvular disease, treatment-related care, and epidemiologic or registry-oriented topics, while primary prevention, implementation research, policy translation, health systems research, and patient-centered approaches appeared less visible. Future RF/RHD research in Southeast Asia should prioritize underrepresented countries and upstream, systems-oriented research domains that can support locally relevant and sustainable disease control.

## Data Availability

The datasets used and/or analyzed during the current study are available from the corresponding author on reasonable request.

## Abbreviations

ASIR: Age–standardized incidence rate
ASPR: Age–standardized prevalence rate
RF: Rheumatic fever
RHD: Rheumatic heart disease
SEA: Southeast Asia
